# The gut microbiota regulates diabetic retinopathy in adult rats

**DOI:** 10.3389/fmicb.2025.1479792

**Published:** 2025-01-29

**Authors:** Jueyu Hou, Zhongping Lv, Yujiao Wang, Danian Chen

**Affiliations:** ^1^Department of Ophthalmology, West China Hospital, Sichuan University, Chengdu, China; ^2^Research Laboratory of Ophthalmology and Vision Sciences, Eye Research Institute, West China Hospital, Sichuan University, Chengdu, China

**Keywords:** diabetic retinopathy, microglia, gut microbiota, broad-spectrum antibiotics, retinal ganglion cells, BN rats

## Abstract

**Introduction:**

Diabetic retinopathy (DR) is the most common complication of diabetes. Neuronal apoptosis, activated microglia, and microvascular changes are early features of DR. The gut microbiota is critical for the maturation and activation of microglia in the brain, and DR patients exhibit gut dysbiosis. However, the effect of the gut microbiota on retinal microglia under normal or diabetic conditions is still unclear.

**Methods:**

Type 2 diabetes (T2D) was established in male adult Brown Norway (BN) rats, and they were treated with gavage of broad-spectrum antibiotic (ABX) suspension. Retinal fundus fluorescein angiography was performed to observe the dynamic growth process and leakage of blood vessels. Retro-orbital injection of FITC-Dextran was performed to observe the changes in blood-retinal barriers. After treatment with ABX and diabetes lasting for more than 6 months, 16S RNA sequencing of stool samples was performed to determine changes in the gut microbiome and mass spectrometry was used to analyze metabolome changes. IBA1, IB4, and Brn3 staining were performed on adult rats’ retinal wholemount or sections to observe the changes in microglia, blood vessels and the number of ganglion cells.

**Results:**

Long-term (6 months) T2D caused gut dysbiosis with increased average taxa numbers. We showed that broad-spectrum antibiotics (ABXs) gavage can reduce the average number of gut microbiota taxa and retinal microglia in adult male BN rats with or without T2D. Interestingly, adult male BN rats with T2D for more than 6 months showed a loss of retinal ganglion cells (RGCs) without significant changes in retinal microglia or retinal vascular vessels. However, ABX gavage reduced retinal microglia and alleviated RGC damage in these T2D rats.

**Conclusion:**

Our data suggests that ABX gavage-induced gut dysbiosis can reduce retinal microglia in adult rats and alleviate RGC loss in long-term T2D rats. Targeting the gut microbiota may be a future therapeutic strategy for DR management.

## 1 Introduction

Diabetic retinopathy (DR) is the most common complication of diabetes. It seriously affects the vision of diabetic patients and is the leading cause of blindness in the working-age population. DR was initially considered a microvascular disease; however, current views agree that early changes in DR also include retinal neurodegeneration and retinal inflammation (Wong et al., 2016). Retinal microglia are essential participants in retinal inflammation. Microglial activation is considered the earliest manifestation of DR inflammation and can occur before Müller cell activation (Zeng et al., 2008; Sorrentino et al., 2016).

Many microorganisms (commensal microbiota) inhabit the mucosal and epidermal surfaces of the human body. Notably, the intestinal tract serves as these microorganisms’ primary site of activity and habitation. The gut microbiota comprises approximately 10^13^ microbial cells. The main gut bacterial phyla include *Firmicutes* (60%), *Bacteroidetes*, and *Actinobacteria* (Szablewski, 2018). The commensal and their hosts are in a mutually beneficial situation in which the commensal microbiota depends on the host for nutrient acquisition and propagation; in turn, the gut microbiota also plays essential roles in host digestion, mineral uptake, vitamin synthesis, drug metabolism, and immune modulation (Li et al., 2019). Dysbiosis represents a prevalent imbalance in the gut microbiota composition (Robles Alonso and Guarner, 2013). Dysbiosis can alter immune regulatory signals, leading to pathological conditions in many organs (Gritz and Bhandari, 2015). Increasing evidence suggests dysbiosis is closely related to many ocular diseases, including DR (Fu et al., 2023). DR patients (Jayasudha et al., 2020; Das et al., 2021; Huang et al., 2021; Ye et al., 2021; Zhou et al., 2021) and DR animal model, such as *db/db* mice (Beli et al., 2018), all have gut dysbiosis. Interestingly, intermittent fasting (IF) can prevent DR by regulating the gut microbiota in *db/db* mice (Beli et al., 2018). The gut microbiota is crucial for the maturation and activation of microglia in the brain (Erny et al., 2015; Thion et al., 2018; Mossad et al., 2022). However, the effect of the gut microbiota on retinal microglia is not fully understood. Whether the gut microbiota plays the same role on normal retinal microglia or microglia under diabetic conditions, as well as whether the gut microbiota can modify DR phenotypes through microglia, are not yet clear.

This study aimed to investigate the effect of gut microbiota on retinal microglia, neurons, and blood vessels in adult diabetic rats and to explore new ideas for DR treatment. We first established a T2D rat model and characterized its gut microbiota changes. We then administered broad-spectrum antibiotics (ABX) by gavage to mimic germ-free (GF) conditions (Bayer et al., 2019; Kennedy et al., 2018). We found that ABX gavage-induced gut dysbiosis could reduce the number of retinal microglia in adult rats. ABX gavage alleviated RGC loss in long-term T2D animals by reducing the number of retinal microglia.

## 2 Materials and methods

### 2.1 Animals

**Figure 1:**
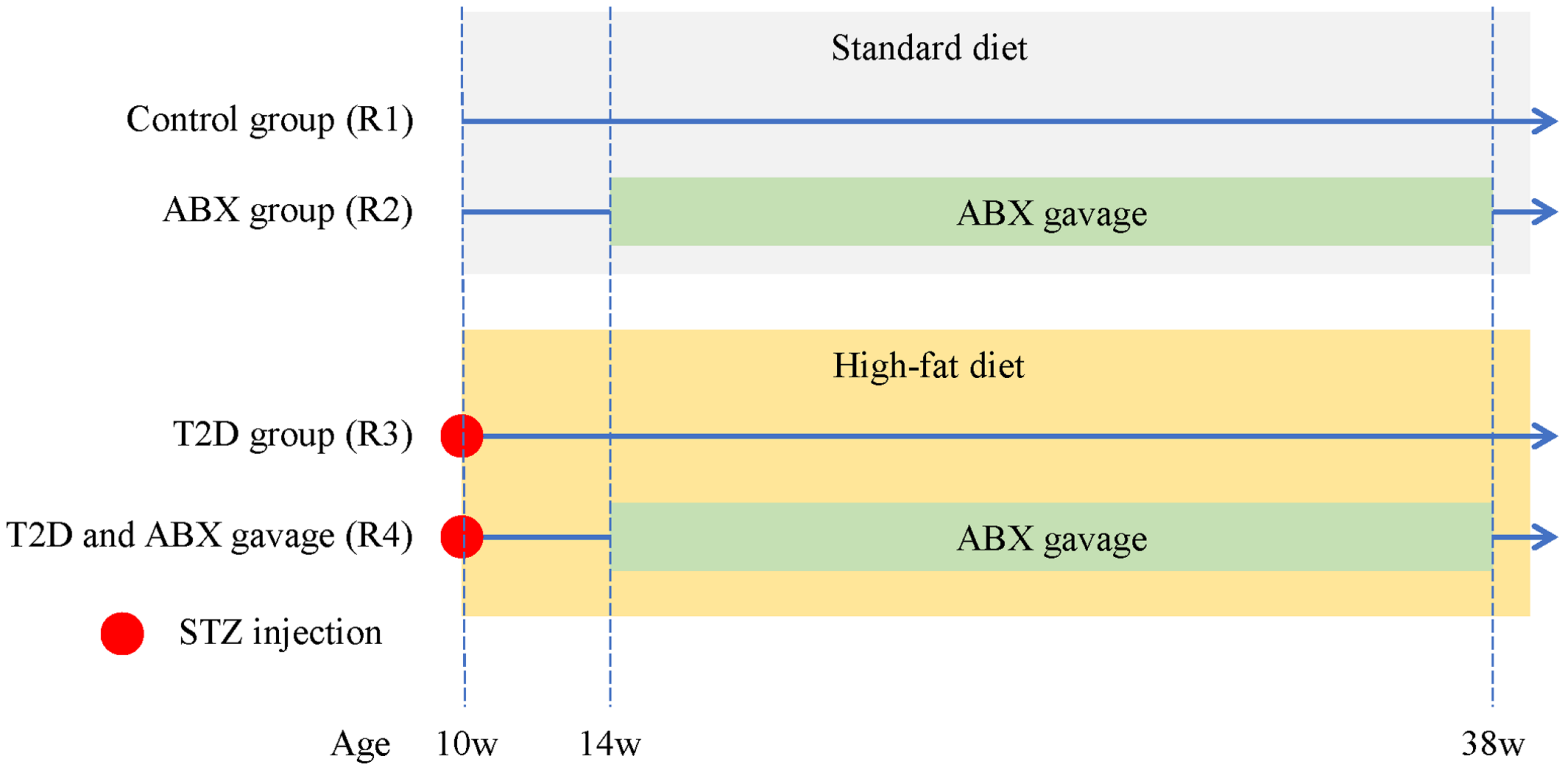
The experimental groups of adult BN male rats. The control group (R1) was fed a standard diet throughout the entire experiment. The ABX group (R2) was fed a standard diet throughout the whole experiment and received ABX via gavage from 14 weeks to 38 weeks of age. T2D rats (R3) received an i.p. injection of STZ at 10 weeks old and were fed a high-fat diet throughout the experiment. T2D/ABX gavage rats (R4) received an i.p. injection of STZ at 10 weeks old. They were also fed a high-fat diet throughout the entire experiment and received ABX via gavage from 14 weeks to 38 weeks of age. ABX, broad-spectrum antibiotics; T2D, type 2 diabetes; STZ, streptozotocin.

Specific pathogen-free (SPF)-grade male Brown Norway (BN) rats (8 weeks old, 200–250 g) were purchased from Beijing Vital River Laboratory Animal Technology Co., Ltd. The animals were raised and bred under SPF conditions. Access to water and standard rat chow was given ad libitum, and the animals were provided with a 12-h dark/12-h light cycle. All procedures used in the animal experiments followed the guidelines of the Association for Research in Vision and Ophthalmology (ARVO) Statement for the Use of Animals in Ophthalmic and Vision Research and by the regulations of the Experimental Animal Ethics Committee of West China Hospital of Sichuan University (AUP# 2018008A). After 2 weeks of acclimation to standard diets, male BN rats were randomly divided into the following four groups (Figure 1), including Group 1 (R1, control, *n* = 4), Group 2 (R2, ABX gavage, *n* = 4), Group 3 (R3, T2D modeling, *n* = 6), and Group 4 (R4, ABX gavage after T2D modeling, *n* = 6).

### 2.2 Inducing type 2 diabetes (T2D)

Streptozotocin (STZ, Sigma–Aldrich Chemical Co., Saint-Quentin Fallavier, France) was freshly prepared in citrate buffer (pH 4.5) (Solarbio, China) to a final concentration of 40 mg/mL. Group 3–4 rats received an i.p. injection of STZ (40 mg/kg body weight) and were subsequently fed a high-fat diet (Beijing Keao Xieli Feed Co., Ltd., D12451). The control group (R1) and ABX gavage group (R2) received equal citrate buffer and were fed standard diets (Figure 1). Animals were weighed, and tail blood was collected for glucose measurement using a standard glucometer (Contour™ Plus, Ascensia).

### 2.3 Antibiotic treatment

Broad-spectrum antibiotic (ABX) treatment is commonly used in gut microbiome research and is considered a standard microbiome depletion protocol. An antibiotic cocktail was delivered to the rats by gavage to deplete the gut microbiota. The antibiotic cocktail was freshly made every day and was composed of five antibiotics, namely, vancomycin (10 mg/mL, MeilunBio), metronidazole (20 mg/mL, MeilunBio), neomycin (20 mg/mL, Solarbio), ampicillin (2 mg/mL, MeilunBio), and amphotericin B (0.2 mg/mL, MeilunBio), based on previous reports (Reikvam et al., 2011; Johnson and Burnet, 2020). This specific mixture has been shown to reduce the fecal bacterial DNA load by 400-fold without causing morbidity (Reikvam et al., 2011; Johnson and Burnet, 2020). The dosage of the ABX cocktail for gavage was 7 mL/kg body weight per day.

### 2.4 Plasma lipid and lipoprotein analysis

Blood was collected from 14-week-old rats (4 weeks after STZ injection). Triglyceride (TG), total cholesterol (TC), high-density lipoprotein (HDL) cholesterol, and low-density lipoprotein (LDL) cholesterol levels were determined using a Cobas8000c702 analyzer (Roche).

### 2.5 Fasting glucose and oral glucose tolerance test (OGTT)

The rats were fasting and water-free for more than 8 h. Fasting blood glucose was measured the next day. Then, the OGTT was performed using a previously described method. In brief, after overnight fasting, all rats received a gavage of 20% glucose solution (10 mL/kg of body weight), and blood samples were taken via the jugular vein 30, 60, 90, 120, 150, and 180 min later. A glucose meter measured blood glucose level (Accu-Check Performa, Roche Diagnostics, China).

### 2.6 Immunofluorescence staining

For immunofluorescence, eyeballs were fixed for 60 min at 4°C in 4% paraformaldehyde, embedded in OCT (TissueTek 4583), frozen at −80°C and cut into 12 μm sections on Superfrost slides. Antigen retrieval was performed as previously described (Chen et al., 2007). The slides were incubated with blocking solution (1% donkey serum and 0.1% Triton X-100 in PBS) for 1 h and then with primary antibodies against Iba-1 (1:500, Wako, 019–19741) and Brn-3 (1:200, Santa Cruz, SC-6062) overnight at 4°C. Vascular endothelial cells were labeled with FITC-IB4 (Sigma, L2895). Primary antibodies or labeled cells were visualized using donkey anti-rabbit and donkey anti-goat antibodies conjugated with Alexa-488 and Alexa-568 (1:1,000; Molecular Probes). Nuclei were counterstained with DAPI (Sigma, D9542) and mounted with Mowiol medium. Finally, the slides were counterstained with 4′6-diamidino-2-phenylindole (DAPI; Sigma Aldrich Corp.) and mounted with Mowiol mounting medium. The negative control was generated by replacing the primary antibodies with PBS.

Eyeballs were enucleated and incubated for 45 min for whole-mount staining with 4% paraformaldehyde in PBS. A circumferential incision was made around the limbus to harvest the retina. The retinas were incubated at 4°C with primary antibodies against Brn3 (Santa Cruz, SC-6026), Iba-1 (1:500, Wako) and FITC-IB4 for 1 days and then with secondary antibodies (donkey anti-goat/rabbit Alexa Fluor 488) for 1 day at 4°C. After briefly washing with PBS, radial cuts were made to divide the retina into four quadrants to flatten the retina, and the flattened retinas were mounted with Mowiol.

### 2.7 Gut microbiota analysis

Fecal pellets from the R1 (control) and R2 (ABX gavage) groups were collected at approximately 9 months of age, and fecal pellets from the R3–R4 groups were collected after 6 months of diabetic modeling (at approximately 9 months of age); all fecal pellets were immediately stored at −80°C until further analysis. DNA was extracted using the OMEGA Soil DNA Kit (M5635-02) (Omega Bio-Tek, Norcross, GA, USA) according to the manufacturer’s instructions. The V3–V4 region of 16S rRNA genes was amplified by PCR. The PCR amplicons were quantified using the Quant-iT PicoGreen dsDNA Assay Kit (Invitrogen, Carlsbad, CA, USA). Metagenomic sequencing was conducted utilizing the Illumina NovaSeq platform with paired-end 2 × 250 bp fragments, and subsequent bioinformatics analysis was performed mainly using the QIIME2 and R packages (v3.2.0) at Suzhou PANOMIX Biomedical Tech Co., Ltd.

### 2.8 Untargeted metabolomic analysis

An appropriate amount of fecal sample from the R1–R5 groups was taken and added to 0.6 mL of methanol containing 2-chlorophenyl alanine (4 ppm), vortexed, and ground. After centrifugation at 12,000 rpm at 4°C for 10 min, the supernatant was collected for liquid chromatography–tandem mass spectrometry detection by using an Ultimate 3,000 UHPLC System (Thermo Fisher Scientific, Waltham, MA, USA) at Suzhou PANOMIX Biomedical Tech Co., Ltd. The raw data was converted to mzXML format through MSConvert in the ProteoWizard software package (v3.0.8789). The metabolites were then identified with the HMDB, Massbank, LipidMaps, MzCloud, and KEGG databases. Subsequently, the multivariate data were analyzed and modeled using Ropels software. Differentially abundant metabolites were screened based on *p* values less than 0.05 and VIP values exceeding 1.

### 2.9 Fundus fluorescein angiography (FFA)

The animals were anesthetized via intravenous injection of soluble sodium pentobarbital (40 mg/kg). Tropicamide (5 mg/mL, Santen Pharmaceutical, Osaka, Japan) was used for pupil mydriasis. The Spectralis HRA + OCT imaging system (Heidelberg Engineering, Heidelberg, Germany) obtained FFA according to the manufacturer’s protocol, as previously reported (Querques et al., 2020; Arrigo et al., 2021). Then, FFA was performed by using 10% sodium fluorescein at 0.03 mL/kg at a rate of 1 mL/s from the tail vein. A series of images were taken at the early phase (30 s) and late phase (5–6 min) to identify retinal vessels.

### 2.10 Retro-orbital injection of FITC-dextran

FITC-dextran (FD2000S; Sigma) was dissolved in ultrapure water at 50 mg/mL. The rats were anesthetized via intravenous injection of soluble sodium pentobarbital (40 mg/kg) and placed in right lateral recumbency with their heads facing to the left. A 27-gauge needle was used at a 45° angle to produce a puncture of 3–4 mm in length into the rat’s orbital venous sinus, and 0.05 mL FITC-dextran was injected into the left orbit according to previous protocols for mouse (Li et al., 2011; Li et al., 2021). Three minutes after the injection, the animals were euthanized, and their eyeballs were enucleated. The enucleated eyeballs were fixed in 4% paraformaldehyde for 30 min at room temperature. Retinas were harvested, and four incisions were made to flatten them on slides. The retinal wholemounts were photographed using Zeiss Axio Imager Z2 fluorescence microscope.

### 2.11 Microscopy, quantification, and statistics

The stained sections and slides were analyzed using a Zeiss Axio Imager Z2 fluorescence microscope and a Nikon A1RMP confocal microscope. ImageJ 1.50b with a cell counter plugin^1^ was used for cell counting following the online guide. The cells positive for Brn3 and Iba-1 were counted manually. At least 2 images per section, 2 sections per retina, and 3 retinas from each group were counted. For Brn3^+^ and Iba-1^+^ cell counting on the wholemount retina, three equivalent areas (each measuring 150 μm × 150 μm) from each quadrant of each retinal wholemount (a total of 12 regions per retina) were selected. All images for cell counting were captured under a fluorescence microscope using a 20× objective lens.

Representative images were analyzed using AngioTool software (NCI) for vascular blood vessel analysis. The data are expressed as the mean ± standard deviation (mean ± SD). All experiments were carried out with *n* = 3–6. The data were compared using Student’s t-test or one-way ANOVA and Bonferroni correction by SPSS 20.0 (IBM, USA) and PRISM 6 (GraphPad, USA). All *p* values were two-sided and considered statistically significant when the values were <0.05. Statistical analysis methods for microbiomes and metabolites are available upon request.

## 3 Results

### 3.1 STZ and a high-fat diet-induced type 2 diabetes

**Figure 2:**
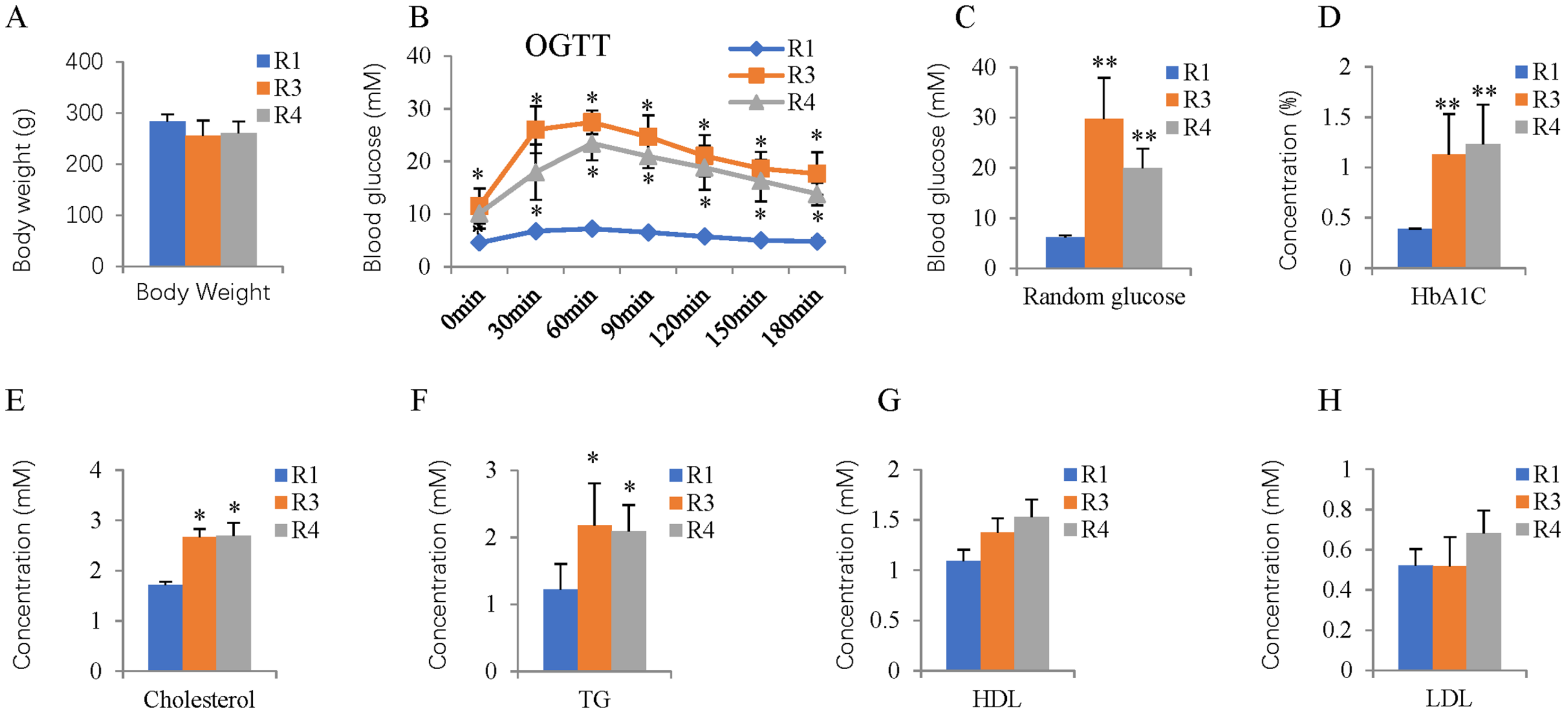
Body weight, OGTT, random glucose, HbA1c, and plasma lipid profiles of the T2D rats. **(A)** Body weight. **(B)** OGTT at 14 weeks of age. **(C)** Random glucose from 14 to 38-week-old mice. **(D–H)** HBA1C, blood cholesterol, TG, HDL, and LDL levels were measured at 14 weeks of age. OGTT, oral glucose tolerance test; TG, triglyceride; HDL, high-density lipoprotein; LDL, low-density lipoprotein. The error bars represent the SDs of the measurements, and the asterisks (*, **) indicate significant differences between control (R1 group) and T2D rats (R3 group) or T2D/ABX rats (R4 group) (*p < 0.05, **p < 0.01, one-way ANOVA followed by Bonferroni correction).

STZ was i.p. injected into male 10-week-old BN rats fed a high-fat diet (Figure 1). Four weeks after the STZ injection, the body weights of the rats in the T2D groups did not change (Figure 2A). The OGTT curves were generally comparable between the T2D (R3 group) and T2D/ABX (R4 group) groups. Still, both groups exhibited a slower glucose clearance rate than the control group, indicating reduced insulin release (Figure 2B). The blood glucose and HbA1c levels in 14-week-old to 38-week-old rats were much higher than those in control rats, indicating successful induction of T2D (Figures 2C,D). Four weeks after STZ injection, the plasma cholesterol and triglyceride (TG) levels were both increased (Figures 2E,F), while the HDL and LDL levels showed no changes (Figures 2G,H).

### 3.2 T2D-induced dysbiosis in BN rats

**Figure 3:**
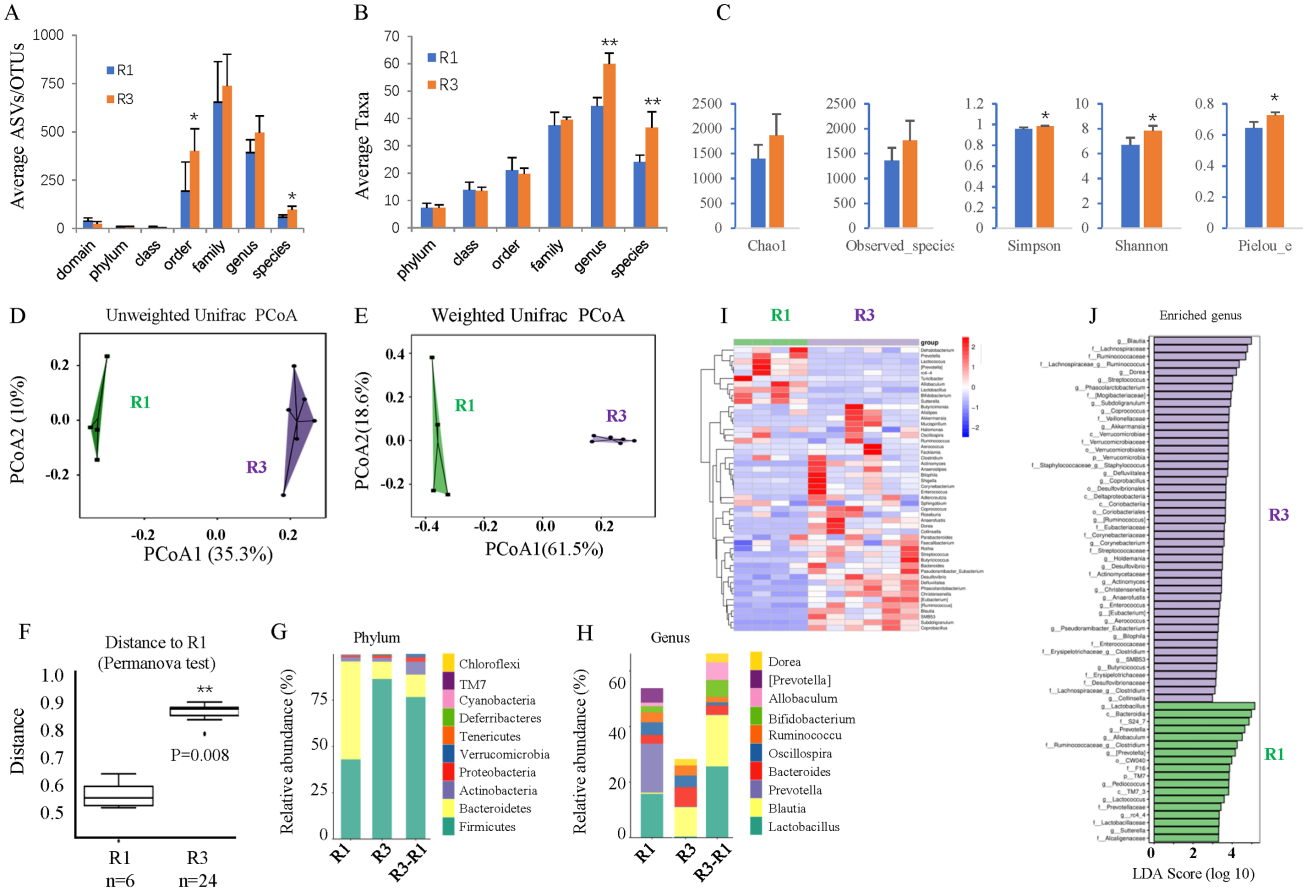
The effects of ABX gavage on the gut microbiota of adult rats. **(A)** The average ASVs/OTUs. **(B)** The average taxa in the control and gavage groups. **(C)** The alpha diversity (Chao1, Observed species, Simpson, Shannon, and Peilou_e indices). **(D)** Unweighted UniFrac PCoA. **(E)** Weighted UniFrac PCoA. **(F)** Permanova test. **(G)** The relative abundances at the phylum level. **(H)** The relative abundances at the genus level. **(I)** Heatmap of the dominant genera. **(J)** LEfSe (LDA effect size) analysis of the enriched genera. ASV, amplicon sequence variant; OTUs, operating taxonomic units. PCoA, principal coordinate analysis. Permanova, permutational multivariate analysis of variance. R1, control group; R2, ABX gavage group.

The gut microbiome was assessed by 16S rDNA sequencing 6 months after T2D induction. T2D for 6 months increased the average ASVs/OTUs (Figure 3A) and taxa (Figure 3B) of the gut microbes, especially at the genus and species levels. Alpha diversity analysis (Figure 3C) revealed no changes in species richness (Chao1 and Observed species indices), increased diversity (Simpson and Shannon indices), and increased evenness (Peilou_e index). Principal coordinate analysis (PCoA) suggested that T2D considerably altered the beta diversity. Both the unweighted (Figure 3D) and weighted (Figure 3E) UniFrac distances showed differences in the structure and composition of the gut bacterial community between the control and T2D groups, which was confirmed by the Permanova test (Figure 3F).

As such, the bacterial composition at the phylum and genus levels was also significantly changed. The abundance of *Bacteroidetes* was greatly reduced at the phylum level, but that of *Firmicutes* was significantly increased in the T2D group; thus, the F/B ratio was increased (Figure 3G). At the genus level, the abundances of *Blautia* and *Bacteroides* were increased, while those of *Lactobacillus* and *Prevotella* decreased in T2D rats (Figure 3H). A clustering heatmap using the abundance data of the top 50 genera showed significant differences between the control and ABX gavage groups (Figure 3I). Based on the LEfSe analysis (LDA), there were 47 dominant genera in the T2D group, while 18 dominant genera were in the control group (Figure 3J).

**Figure 4:**
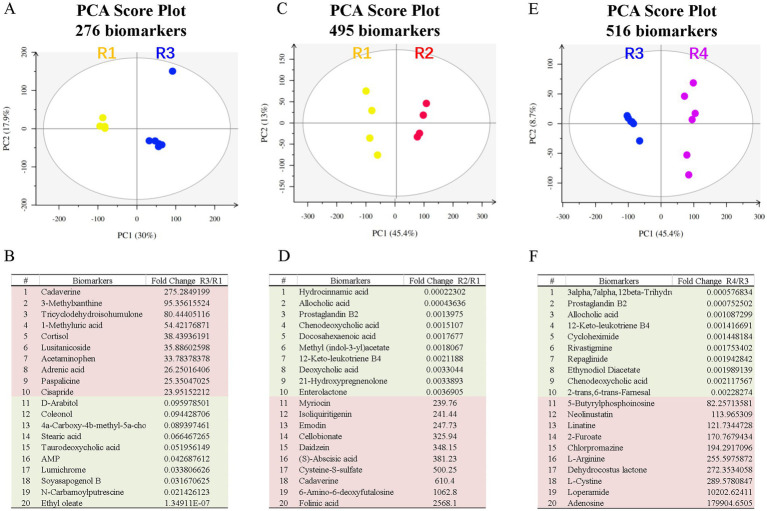
Fecal metabolite profiling of the R1-R5 groups. The PCA score plots and top metabolite biomarkers are shown for the R2/R1 **(A,B)**, R3/R1 **(C,D)**, and R4/R3 **(E,F)** groups. Downregulated biomarkers are shown in green blocks, and upregulated biomarkers are in pink blocks. PCA, principal component analysis.

We also performed untargeted metabolomic analysis on fecal samples from these rats. PCA revealed significant differences between the control and T2D groups (Figure 4A) and identified 276 metabolite biomarkers (Supplementary Table S1), including 6 SCFAs (Supplementary Table S2). The top 10 increased metabolites and decreased metabolites are shown in Figure 4B.

**Figure 5:**
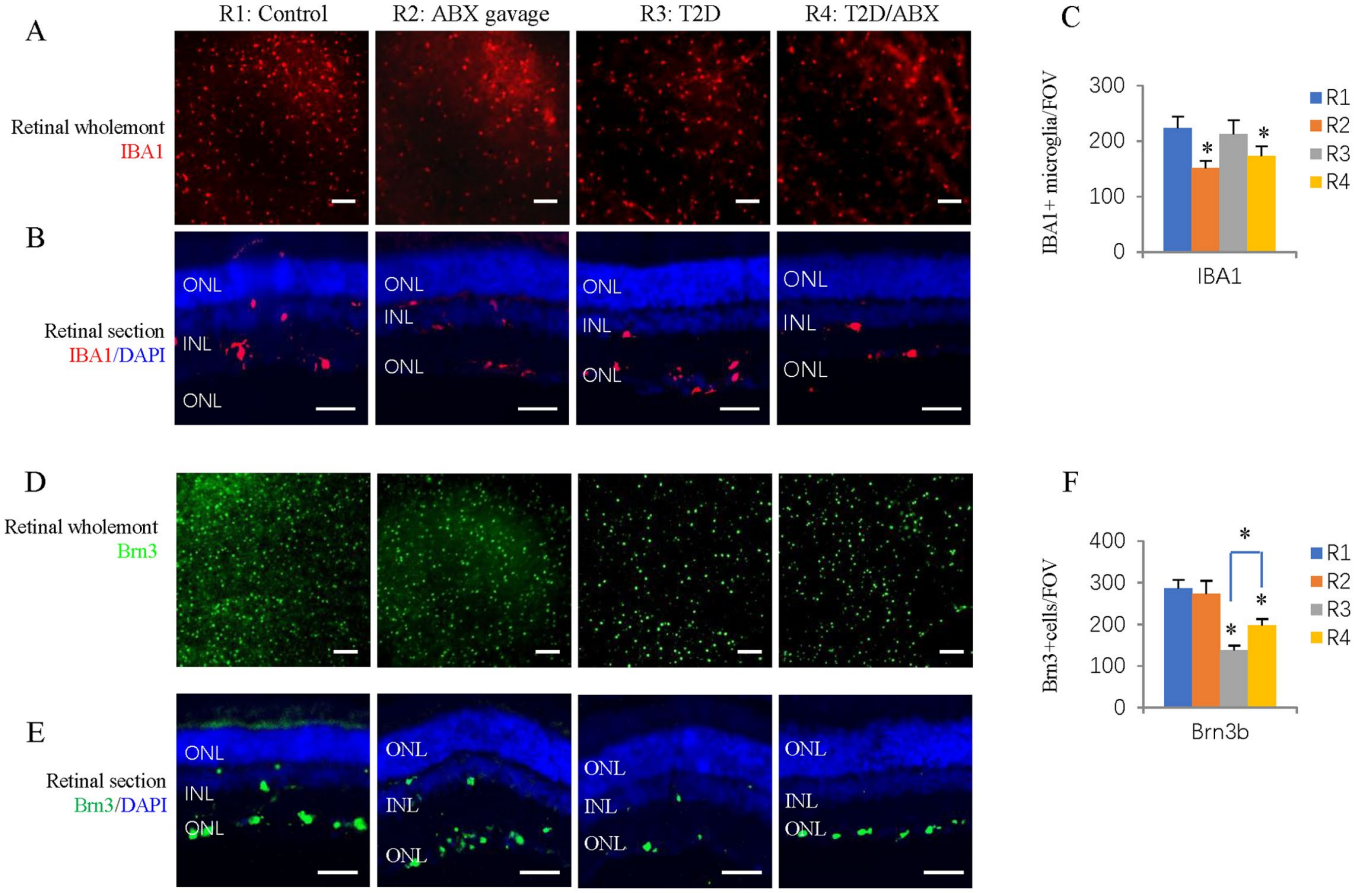
The effects of ABX gavage and long-term T2D on retinal microglia and ganglion cells. **(A)** Retinal wholemounts of 38-week-old rats from the indicated groups were stained for microglia (IBA1, red). **(B)** Horizontal retinal sections of 38-week-old rats from the indicated groups were stained for the nuclei (DAPI, blue) and microglia (IBA1, red). **(C)** Quantification of IBA1+ cells in the retinal wholemounts. **(D)** Retinal wholemounts of 38-week-old rats from the indicated groups were stained for ganglion cells (Brn3, green). **(E)** Horizontal retinal sections of 38-week-old rats from the indicated groups were stained for nuclei (DAPI, blue) and ganglion cells (Brn3, green). **(F)** Quantification of Brn3+ cells in the retinal wholemounts. The error bars represent the SDs of the measurements, and the asterisks (*, **) indicate significant differences between the control group (R1 group) and the other groups, including the ABX gavage group (R2), T2D group (R3), and T2D/ABX group (R4) (*p < 0.05, **p < 0.01; one-way ANOVA followed by Bonferroni correction). The scale bars are 200 μm **(A,D)** and 50 μm **(B,E)**. ONL, outer nuclear layer; INL, inner nuclear layer; GCL, ganglion cell layer.

**Figure 6:**
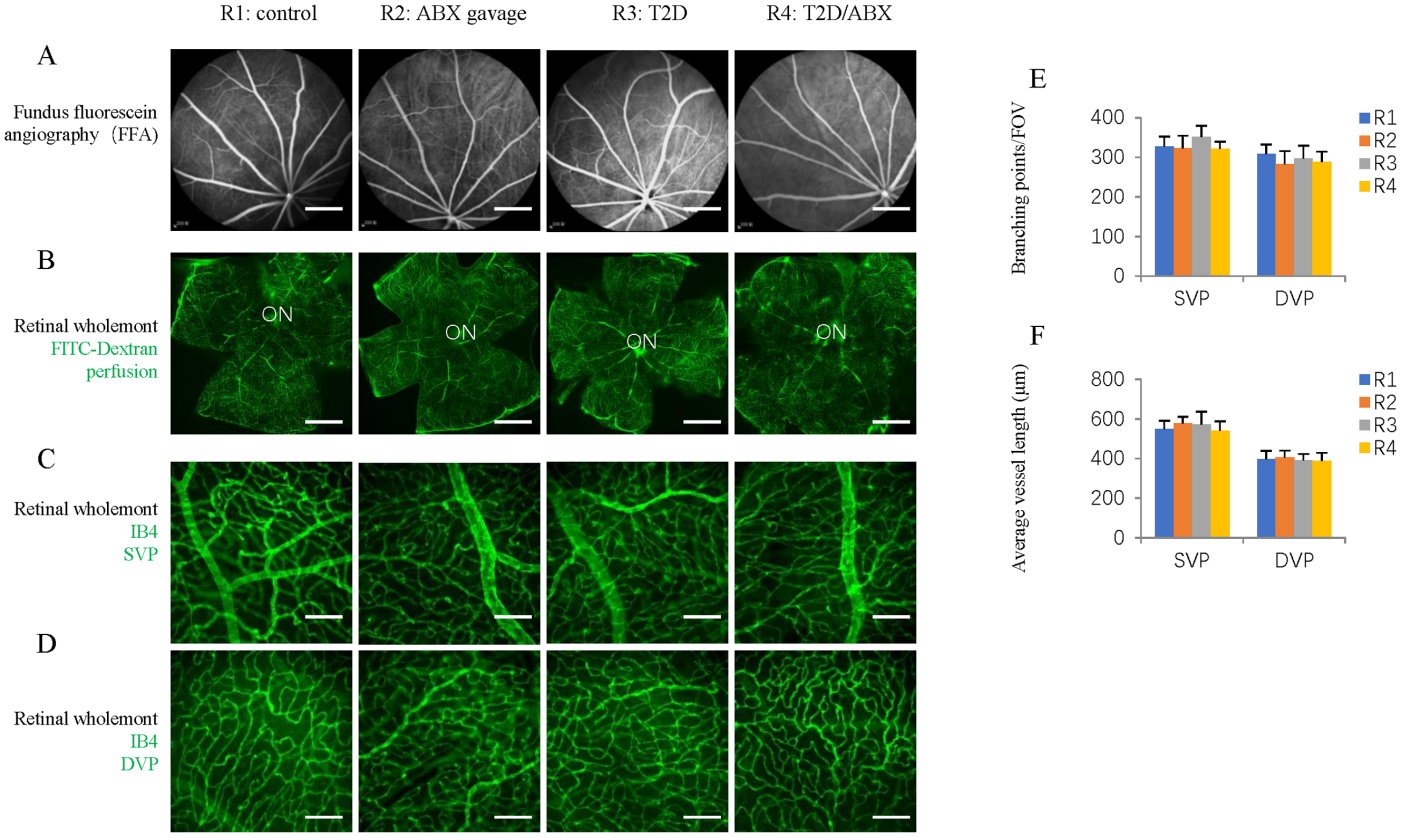
The effects of ABX gavage and long-term T2D on retinal blood vessels. **(A)** Images of ocular fundus from 38-week-old rats in the indicated groups in the late stage of FFA. **(B)** Retinal wholemounts of 38-week-old rats from the indicated groups received retro-orbital injection of FITC-Dextran (FITC-Dextran, green). **(C)** Retinal wholemounts of 38-week-old rats from the indicated groups were stained for vascular vessels (IB4, green). These are SVPs. **(D)** Retinal wholemounts of 38-week-old rats from the indicated groups were stained for vascular vessels (IB4, green). These are DVPs. **(E)** Quantification of the branching points of retinal blood vessels. **(F)** Quantification of the average vessel length of retinal blood vessels. The error bars represent the SDs of the measurements, and the asterisks (*, **) indicate significant differences between the control group (R1 group) and the other groups, including the ABX gavage group (R2), T2D group (R3), and T2D/ABX group (R4) (*p < 0.05, **p < 0.01; one-way ANOVA followed by Bonferroni correction). The scale bars are 600 μm **(A,B)** and 50 μm **(C,D)**. FFA, fundus fluorescein angiography; ON, optic nerve; SVP, superficial vascular plexus; DVP, deep vascular plexus.

It is known that long-term T2D can induce DR, including retinal degeneration, inflammation, and microvascular defects (Zeng et al., 2000). We looked at these features in our T2D rat model. Retinal wholemount and section staining showed that the number and retinal distribution of IBA1^+^ retinal microglia did not change in the T2D group (Figures 5A–C). However, T2D significantly reduced the number of retinal ganglion cells (Figures 5D,E). Even though T2D persisted for more than 6 months, fundus fluorescein angiography (FFA) and FITC-Dextran perfusion by retro-orbital injection did not reveal any vascular leakage (Figures 6A,B), and IB4 staining did not reveal significant changes in retinal blood vessels (Figures 6C–F). Thus, 6 months after the onset of T2D, the substantial change in the retina was ganglion cell loss, supporting the notion that retinal degeneration is the earliest change of DR (Sachdeva, 2021).

Gut dysbiosis and metabolite changes may contribute to the reduction in retinal ganglions. To test this hypothesis, we investigated if ABX (broad-spectrum antibiotics treatment) can affect rat microbiota and retinal phenotypes in control rats.

### 3.3 ABX gavage depleted the gut microbiota of adult BN rats and reduced retinal microglia

**Figure 7:**
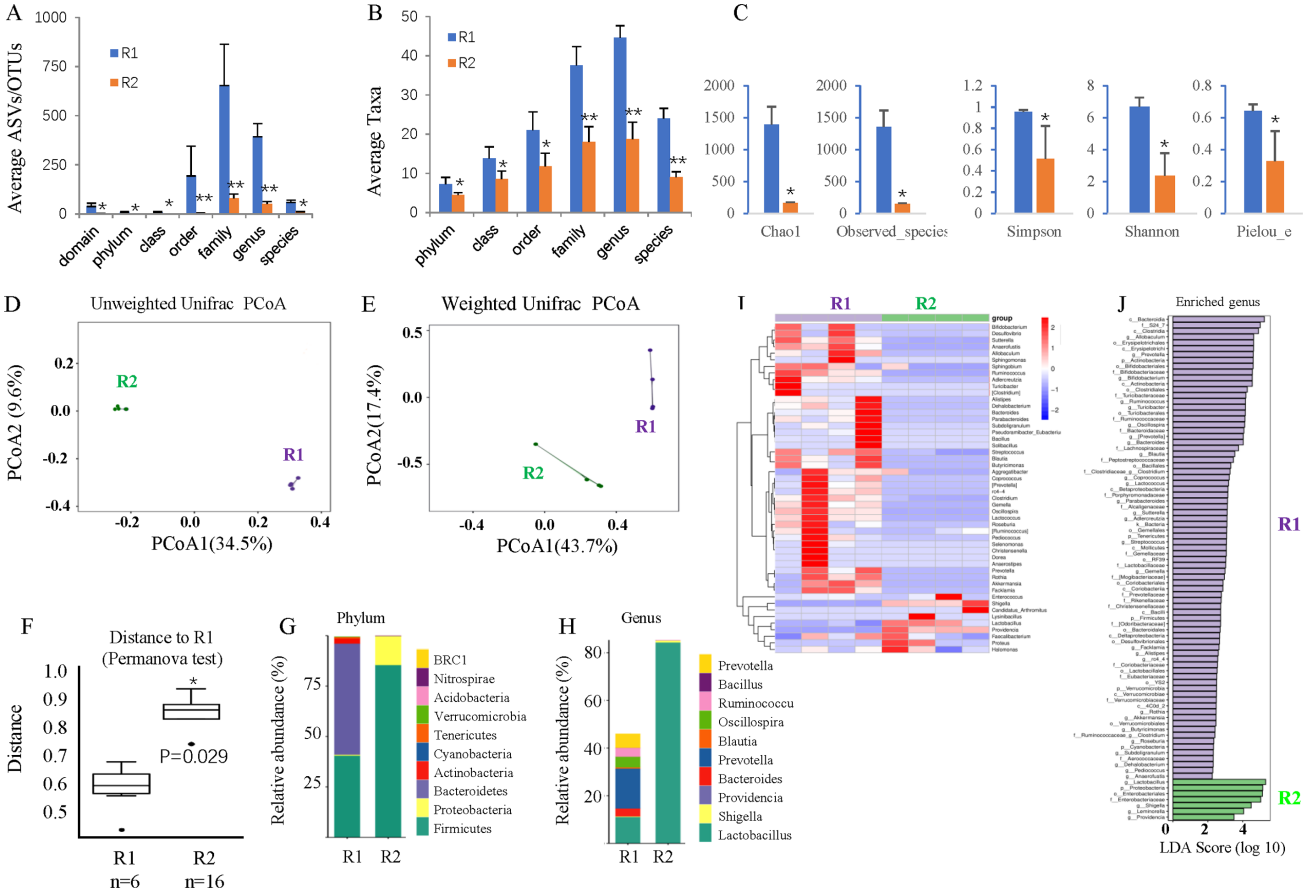
The effects of long-term T2D on the gut microbes of adult rats. **(A)** The average ASVs/IPs. **(B)** The average taxa in the control and gavage groups. **(C)** The alpha diversity (Chao1, Observed species, Simpson, Shannon, and Peilou_e indices). **(D)** Unweighted UniFrac PCoA. **(E)** Weighted UniFrac PCoA. **(F)** Permanova test. **(G)** The relative abundances at the phylum level. **(H)** The relative abundances at the genus level. **(I)** Heatmap of the dominant genera. **(J)** LEfSe (LDA effect size) analysis of the enriched genera. ASV, amplicon sequence variant; OTUs, operating taxonomic units. PCoA, principal coordinate analysis. Permanova, permutational multivariate analysis of variance. R1, control group; R3, T2D group.

Like mice, gavage of the ABX cocktail depleted the gut microbiota of adult rats. 16S rDNA sequencing revealed that ABX gavage for 6 months significantly reduced the average ASVs/OTUs (Figure 7A) and taxa (Figure 7B) of the gut microbes, especially at the family, genus, and species levels. Alpha diversity analysis (Figure 7C) revealed significantly reduced richness (Chao1 and Observed species indices), reduced diversity (Simpson and Shannon indices), and reduced evenness (Peilou_e index). PCoA suggested that the beta diversity was significantly altered by ABX gavage. Both the unweighted (Figure 7D) and weighted (Figure 7E) UniFrac distances showed differences in the structure and composition of the gut bacterial community between the control and ABX gavage groups, which was confirmed by the Permanova test (Figure 7F).

The bacterial composition at the phylum and genus levels was significantly changed. At the phylum level, there were almost no *Bacteroidetes* but many more *Firmicutes* in the ABX gavage group; thus, the *Firmicutes/Bacteroidetes* (F/B) ratio increased (Figure 7G). At the genus level, *Lactobacillus* accounted for more than 80% of the bacteria in the ABX-treated group (Figure 7H). A clustering heatmap using the abundance data of the top 50 genera showed significant differences between the control and ABX gavage groups (Figure 7I). Linear discriminant analysis (LDA) effect size (LEfSe) revealed that there were only 7 dominant genera in the ABX gavage group, while there were 79 dominant genera in the control group (Figure 7J).

Because the gut microbiota can affect microglia in the brain, we examined the number and distribution of retinal microglia in these adult rats. Retinal wholemount and section staining revealed significantly reduced IBA1^+^ microglia in the ABX gavage group (Figures 5A–C). Section staining indicated that the distribution of retinal microglia had not been changed; they were in the ganglion cell layer, inner nuclear layer, and outer plexiform layers (Figure 5B). However, Retinal wholemount and section staining of Brn3 suggested that ABX gavage did not considerably affect ganglion cells (Figures 5D–F). Furthermore, FFA and FITC-Dextran perfusion did not detect vascular leakage in either group (Figures 6A,B), and retinal wholemount staining of IB4 found no changes in vessel branching points and vessel length between R1 and R2 groups (Figures 6C–F).

Principal component analysis (PCA) on untargeted metabolomic data revealed significant differences between the control and ABX gavage groups (Figure 4C), and 495 metabolite biomarkers were identified (Supplementary Table S1), including 12 SCFAs (Supplementary Table S2). The top 10 decreased and increased metabolites are shown in Figure 4D. These metabolites may contribute to the reduction in retinal microglia.

### 3.4 ABX gavage reduced retinal microglia and rescued retinal ganglion cell loss in T2D BN rats

**Figure 8:**
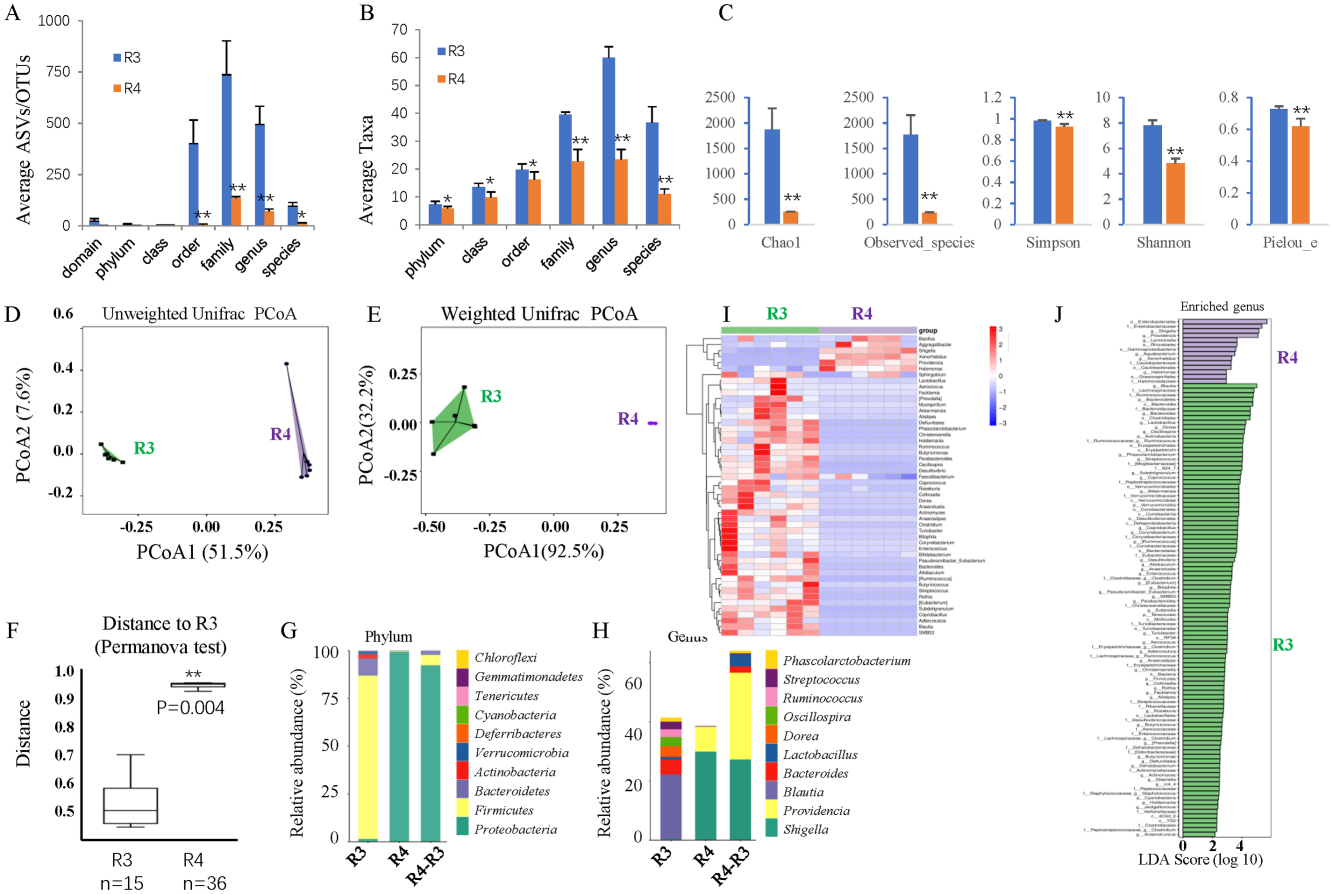
The effects of ABX gavage on the gut microbes of adult T2D rats. **(A)** The average ASVs/OTUs. **(B)** The average taxa in the control and gavage groups. **(C)** The alpha diversity (Chao1, Observed species, Simpson, Shannon, and Peilou_e indices). **(D)** Unweighted UniFrac PCoA. **(E)** Weighted UniFrac PCoA. **(F)** Permanova test. **(G)** The relative abundances at the phylum level. **(H)** The relative abundances at the genus level. **(I)** Heatmap of the dominant genera. **(J)** LEfSe (LDA effect size) analysis of the enriched genera. ASV, amplicon sequence variant; OTUs, operating taxonomic units. PCoA, principal coordinate analysis. Permanova, permutational multivariate analysis of variance. R3, T2D group; R4, T2D/ABX gavage group.

To test whether dysbiosis contributes to retinal ganglion cell loss in T2D rats, we gavaged T2D rats with ABX for approximately 6 months. 16S rDNA sequencing revealed that ABX gavage significantly reduced the gut microbes’ average ASVs/OTUs (Figure 8A) and taxa (Figure 8B), especially at the family, genus, and species levels. Alpha diversity analysis (Figure 8C) revealed significantly reduced species richness (Chao1 and Observed species indices), reduced diversity (Simpson and Shannon indices), and reduced evenness (Peilou_e index). PCoA suggested that the beta diversity was significantly altered by ABX gavage. Both the un-weighted (Figure 8D) and weighted (Figure 8E) UniFrac distances showed differences in the structure and composition of the gut bacterial community between the T2D and T2D/ABX gavage groups, which was confirmed by the Permanova test (Figure 8F).

As such, the bacterial composition at the phylum and genus levels was also significantly changed. At the phylum level, the abundances of both *Bacteroidetes* and *Firmicutes* were decreased, and the F/B ratio was reduced (Figure 8G). Proteobacteria was the most dominant phylum in the T2D/ABX-treated rats (Figure 8G). At the genus level, the abundances of *Shigella* and *Providencia* were increased in the T2D/ABX-gavage rats (Figure 8H). A clustering heatmap using the abundance data of the top 50 genera showed significant differences between the T2D and T2D/ABX gavage groups (Figure 8I). LEfSe revealed only 14 dominant genera in the T2D/ABX gavage group, while 99 dominant genera in the T2D group (Figure 8J).

We examined whether ABX gavage affects retinal phenotypes in T2D rats. Retinal wholemount and section staining revealed substantially fewer IBA1^+^ microglia in the T2D/ABX gavage group than in the T2D group (Figures 5A–C). Interestingly, ABX gavage rescued the ganglion cell loss in the T2D group, but the number of retinal ganglion cells was still lower than that in the control group (Figures 5D,E). The T2D rats had no major vascular defects, so ABX gavage did not affect the retinal blood vessels of these T2D rats based on FFA, FITC-Dextran perfusion, and IB4 staining (Figures 6A–F).

We performed untargeted metabolomic analysis on fecal samples to understand how ABX gavage rescued the ganglion cell loss in T2D rats. PCA revealed significant differences between the T2D and T2D/ABX gavage groups (Figure 4E), and 516 metabolite biomarkers were identified (Supplementary Table S1), including 14 SCFAs (Supplementary Table S2). The top 10 decreased metabolites and increased metabolites are shown in Figure 4F. These metabolites may contribute to rescuing retinal ganglion cells in T2D rats.

Comparing these metabolites, we identified the top 10 metabolites common among R2/R1, R3/R1, and R4/R3 groups (Table 1). All these metabolites increased in T2D rats but decreased in the ABX gavage groups (R1 and R4), including Cycloheximide, Chenodeoxycholic acid (CDCA), Tricyclodehydroisohumulone (TCD), palmitoleic acid, and others. Some of these metabolites may activate retinal microglia and reduce retinal ganglion cells in T2D rats.

**Table 1:** Top 10 common metabolite markers among R1–R4 groups.

#	Metabolite biomarkers	log2(FC_R2/R1)	log2(FC_R3/R1)	log2(FC_R4/R3)
1	Cycloheximide	−7.82	2.48	−9.43
2	Chenodeoxycholic acid (CDCA)	−9.37	2.22	−8.88
3	Tricyclodehydroisohumulone (TCD)	−4.83	6.33	−8.69
4	1-Methyl-6-phenyl-1H-imidazo[4,5-b] pyridin-2-amine (PhIP)	−5.42	2.34	−8.05
5	6-Keto-prostaglandin F1a	−4.27	2.32	−5.95
6	13S-hydroxyoctadecadienoic acid	−2.02	4.31	−5.82
7	5a-Pregnane-3,20-dione	−3.58	2.18	−4.56
8	Mesobilirubinogen	−6.29	3.09	−4.39
9	Palmitoleic acid	−2.14	3.05	−4.28
10	(R,S)-Norlaudanosoline	−2.70	2.76	−4.22

R1: Control group; R2: ABX gavage group; R3: T2D rats; R4: T2D/ABX gavage rats; FC: fold changes.

## 4 Discussion

### 4.1 DR and gut dysbiosis

Diabetic retinopathy (DR) is the most common diabetes complication and the leading cause of blindness in the working-age population. Both DR patients (Jayasudha et al., 2020; Das et al., 2021; Huang et al., 2021; Ye et al., 2021; Zhou et al., 2021) and DR animal models, such as *db/db* mice (Beli et al., 2018) and Akita mice (Prasad et al., 2022), exhibit gut dysbiosis. Gut dysbiosis may be necessary for DR development (Fu et al., 2023). This study found that long-term T2D can induce gut dysbiosis, with increased taxa number, alpha diversity, and *Firmicutes/Bacteroidetes* (F/B) ratio. At the genus level, the abundances of *Blautia* and *Bacteroides* increased, while those of *Lactobacillus* and *Prevotella* decreased.

Our rat model is different from that of human T2D patients, as previous studies have shown reduced diversity of the gut microbiota in T2DM patients, with a decrease in the abundance of *Firmicutes* and the F/B ratio (Huang et al., 2021), similar to the gut microbiota in aged humans (Mäkivuokko et al., 2010; Fernandes et al., 2019). Thus, the gut microbiota in the male BN rat model of T2DM we established may differ from the gut microbiota of human T2DM patients due to the relatively young age of the rats (12 months old, analogous to 20- to 30-year-old humans) (Sengupta, 2013).

There are many studies regarding how gut microbiota influences retinal health or retinal diseases (Fu et al., 2021; Fu et al., 2023; Huang et al., 2023; Serban et al., 2023). Gut microbiota influences retinal health in multiple ways and may represent future therapeutic targets in DR (Serban et al., 2023), such as destruction of intestinal barrier (leaky gut) and microbial metabolites, including SCFAs, TUDCA and lactic acid.

### 4.2 DR features in BN rats

Previous studies conducted using different rat strains have detected DR lesions at different durations of diabetes (Robinson et al., 2012). For instance, after 3 months of diabetes, inflammatory changes in the diabetic rat retina are highly strain dependent, which are detected only in Sprague–Dawley (SD) rats but not Brown Norway (BN), or Long-Evans rats (Kirwin et al., 2009). After 8 months of diabetes, Lewis rats showed the most accelerated loss of retinal vessels and RGCs, whereas Wistar rats showed degeneration of the retinal vascular vessels without significant neurodegeneration and SD rats showed no lesions at this time point (Kern et al., 2010). In this study, long-term (6 months) T2D induced retinal ganglion cell loss but did not increase the number of retinal microglia or alter the retinal vasculature. This may suggest that retinal degeneration is the earliest feature of DR, which is consistent with previous reports (Sachdeva, 2021). Metabolomic analysis revealed 276 differentially abundant metabolites in the T2D group, some of which can be regulated by ABX gavage, such as cycloheximide, chenodeoxycholic acid (CDCA), and palmitoleic acid. If these metabolites play a role in the development of DR, further investigation is needed.

### 4.3 Gut dysbiosis and microglia

Microglia is an immune cell in the brain that plays important roles in modulating inflammation and neurogenesis. The relationship between the gut microbiota and microglia has been a research hotspot in recent years (Mossad and Erny, 2020; Bettag et al., 2023). Several studies have investigated the correlation between the gut microbiota and brain microglia in embryonic, offspring, and adult mice, primarily utilizing germ-free (GF) mice (Erny et al., 2015; Mosher and Wyss-Coray, 2015; Thion et al., 2018). Microglia in GF mice exhibit increased cell density, likely due to the upregulation of Csf1r, Ddit4, and Tgf-ß1, which can promote cell survival and proliferation (Mossad and Erny, 2020). They also have a hyper-ramified morphology and defective function, as they fail to induce an immune response upon the lipopolysaccharide (LPS) challenge. These defects can be partially reversed by supplementation with short-chain fatty acids (SCFAs). Defective microglial morphology and functional phenotypes are also observed in mice treated with broad-spectrum antibiotics (ABXs) for 4 weeks, with an average microglial density of Erny et al. (2015).

In this study, we found that ABX gavage for 6 months can deplete the gut microbiota and reduce the density of retinal microglia, which is slightly different from previous observations in ABX-treated mice (Erny et al., 2015; Cordella et al., 2021). One possible explanation is that our treatment duration was much longer (6 months) than the 2 or 4-week duration used in previous studies.

In adult rats, the gut microbiota mainly affects the recruitment of retinal microglia through metabolites. Metabolomic analysis revealed 495 differentially abundant metabolites including 12 SCFAs in the ABX gavage group (Supplementary Tables S1, S2). The level of palmitoleic acid, which can promote the activation and proliferation of microglia (Wang et al., 2012; Urso and Zhou, 2021), was significantly decreased in ABX gavage-treated rats. This may partially explain ABX-induced microglia reduction.

### 4.4 ABX reduces retinal microglia and RGC loss of BN diabetic rats

Microglia cells are the resident immunocompetent cells in the retina. There are many studies regarding microglia function and retinal health (Guo et al., 2022; Murenu et al., 2022; Rathnasamy et al., 2019). In brief, retinal microglia contribute to the development and function of retina; In the developing retina, they are involved in the pruning of neuronal and vascular networks through the phagocytic removal of dead cell debris (Fernandes et al., 2014). In the adult retina, they reside in the plexiform layers and can secret neurotrophic factors to support retinal cell survival. They continuously monitor their environment and when activated, they shift toward an amoeboid morphology (Davis et al., 1994). Microglial activation is detrimental to normal functioning of the retina and indicative of a variety of retinal diseases. Microglia cells, with their highly motile processes extending into the capillary wall, are likely the first detector of metabolic changes in diabetes (Graeber et al., 2011). Once activated, microglia become mobile and migrate to the site of inflammation and will produce a wide range of pro-inflammatory cytokines, glutamate, ROS, nitrous oxide (NO) and proteases. Under chronic activation conditions, these products can be very toxic to RGCs, inducing retinal dysfunction (Zeng et al., 2008). Thus, activated retinal microglia are a therapeutic target in retinal diseases (Rathnasamy et al., 2019).

Using the same strategy in control rats, we treated T2D rats with ABX via gavage for 6 months. We found that ABX gavage reduced the density of retinal microglia and partially rescued ganglion cell loss in these T2D rats. The mechanism may be attributed to multiple factors, including a decline in the phagocytic function of microglia toward ganglion cells (Bodeutsch and Thanos, 2000; Schafer et al., 2012; Au and Ma, 2022), a reduction in the secretion of proinflammatory factors (Takeda et al., 2018), and potentially the influence of gut microbiota metabolites.

Metabolomic analysis revealed 516 differentially abundant metabolites including 14 SCFAs in the T2D/(T2D + ABX) group. Notably, microglia-activator palmitoleic acid increased in the T2D rats but greatly reduced in the ABX-treated T2D rats. Benzene-induced hematopoietic toxicity was mediated by the gut microbiota-palmitoleic acid axis (Zhang et al., 2024). If this axis mediates T2D-induced ganglion cell loss, it deserves further investigation.

Even though ABX gavage can partially rescue RGC loss in these T2D rats, it may be not practical to apply ABX to treat T2D patients, as known side effects (Wiens et al., 2018). Diabetes-induced dysbiosis can be treated by probiotics, or fecal microbiota transplantation (FMT) (Fu et al., 2023).

### 4.5 Limitations

There are some limitations of this study. First, we only measured the density and distribution of retinal microglia but not their morphology and function in the T2D rat model. Second, even though we had determined some candidate metabolites, we had not measured their plasma concentration and tested whether they were responsible for the retinal phenotypes we observed.

## 5 Conclusion

For more than 6 months, T2D in adult male BN rats led to dysbiosis and a significant reduction in retinal ganglion cells but without major changes in retinal microglia or retinal vascular vessels. Broad-spectrum antibiotic (ABX) gavage for 6 months depleted gut microbes in adult rats, reduced retinal microglia and rescued the ganglion loss in T2D rats. The gut microbiota and retinal microglia are future targets for diabetic retinopathy therapy.

## Data Availability

Initial 16S RNA sequences are available in the NCBI Genbank under BioProject PRJNA1208557 and accession numbers SRR31944784-31944803.

## Footnotes

^1^ https://imagej.nih.gov/ij/
